# Supplementary material for: How can the use of different modes of survey data collection introduce bias? An introduction to mode effects using directed acyclic graphs (DAGs)
Source: Am J Epidemiol. 2026 Jan 23;195(5):1406–15. doi: 10.1093/aje/kwag017 (PMC13149020; doi:10.1093/aje/kwag017)

**Title:** How can the use of different modes of survey data collection introduce bias? An introduction to mode effects using directed acyclic graphs (DAGs)

**Authors:** Georgia D Tomova, Richard J Silverwood, Peter WG Tennant, Liam Wright

**Supplementary materials:** Figure S1, Figure S2

**Figure S1. A directed acyclic graph depicting M-bias due to the presence of unobserved common causes of the exposure, outcome, and mode selection.** Shared variation is depicted in green; conditioned nodes are depicted in red and with square brackets. **A)** considers smoking and physical activity as example exposure and outcome, where **Smoking*** and **Physical activity*** are measured versions of the latent **Smoking** and **Physical activity**. The latent exposure and outcome are assumed not to cause **Mode** selection. Unobserved variables **U_1_** and **U_2_** include any common causes of the exposure and outcome, respectively, and mode selection, e.g. socio-economic position, physical disability, alcohol intake. **B)** shows the consequences of naively conditioning on **Mode**, which will open the path **Smoking* -> Smoking -> U_1_ -> Mode <- U_2_ <- Physical activity <- Physical activity***, introducing M-bias, a type of collider bias which can occur even when the latent exposure and outcome themselves are not direct causes of mode selection.


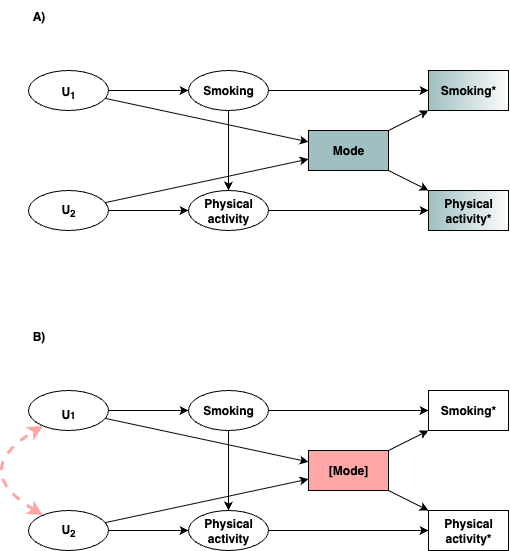


**Figure S2. A directed acyclic graph depicting selection into the sample influenced by the survey modes offered.** It considers the effect of education on anxiety, where **Education*** and **Anxiety*** are measured versions of the latent **Education** and **Anxiety. Mode** may influence selection into the **Sample** if the availability of modes drives any participants to non-respond. The **Sample** is conditioned on (depicted in red and with square brackets) because it is restricted to only those for which data are available for analysis. In this regard, **Mode** itself is also always restricted to the set of modes available to respondents. This will open biasing paths via the common causes of selection into the **Mode** and the **Sample**, including the latent exposure and outcome and any other unobserved common causes (**U**).


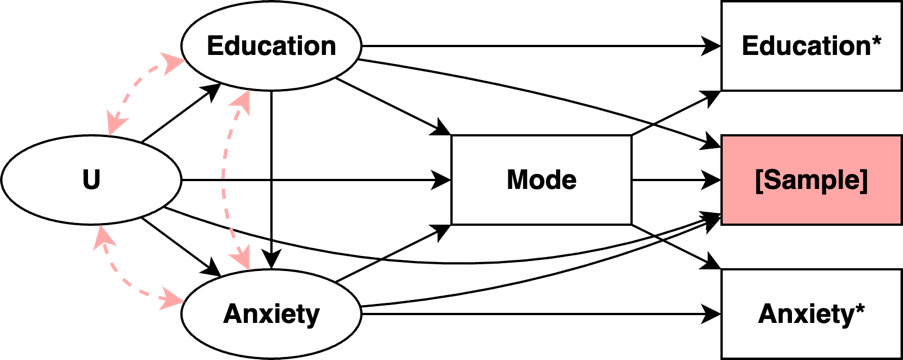

Supplement: Web_Material_kwag017 [file web_material_kwag017.zip › AJE-01214-2025_R2_Supplementary.docx]
